# Efficient and biocompatible new palladium-supported boehmite nanoparticles: synthesis, characterization and application in Suzuki–Miura and Mizoroki–Heck coupling reactions

**DOI:** 10.1039/d3na00403a

**Published:** 2023-08-14

**Authors:** Zahra Hajighasemi, Ali Nahipour, Arash Ghorbani-Choghamarani, Zahrra Taherinia

**Affiliations:** a Department of Chemistry, Faculty of Science, Ilam University Po. Box 69315-516 Ilam Iran a.naghipour@ilam.ac.ir; b Department of Organic Chemistry, Faculty of Chemistry, Bu-Ali Sina University Hamedan 6517838683 Iran a.ghorbani@basu.ac.ir

## Abstract

Palladium complex-supported on boehmite (Pd(0)-SMTU-boehmite) nanoparticles were synthesized and characterized by using XRD, SEM, EDS, TGA, BET, ICP and FT-IR techniques. When applied as a new catalyst for C–C coupling reactions of Suzuki–Miyaura and Mizoroki–Heck in PEG-400 solvent, the Pd(0)-SMTU-boehmite nanoparticles showed excellent activity and recyclability. The study of palladium leaching by the ICP-OES technique and hot filtration led to the catalyst exhibiting excellent stability and recyclability.

## Abstract 2

Immobilized palladium on boehmite nanoparticles effective in Heck and Suzuki cross-coupling reactions.

## Graphical abstract

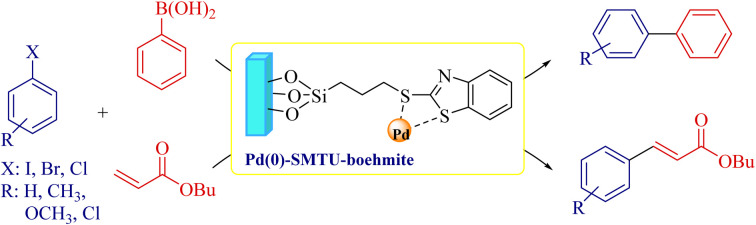

## Introduction

Boehmite nanoparticles are one of the important substrates that are used as heterogeneous catalysts due to their greater stability and efficiency, availability, lower cost and toxicity, better corrosion resistance, and biocompatibility.^1–6^ These nanoparticles contain cubic aluminum oxide hydroxide and their entire surface is capped with hydroxy groups. In the structure of boehmite there are two plates with an octahedral structure in which aluminum ions are placed in the center and surrounded by six oxygen atoms.^7–10^ Carbon–carbon bond formation through a cross-coupling reaction is extensively used in the synthesis of natural compounds and biologically active compounds. The development and expansion of efficient, useful and biologically active compounds is one of the goals of green chemistry today. The development and expansion of efficient and useful carbon–carbon bond formation methods is a performance character in the synthesis of organic compounds.^11–19^ Some of the most frequently used reactions of carbon–carbon bond formation in organic synthesis are Mizoroki–Heck, Sonogashira, Suzuki–Miyaura, Stille, Negishi, *etc.*^20–30^ Among them, the Suzuki reaction is known as the main and general process in the synthesis of biaryls, and palladium compounds usually catalyze this reaction, which includes phenylboronic acid or its derivatives coupling with various aryl halides.^31,32^ Also, one of the most important and generally used reactions in the field of carbon–carbon bond formation is the Mizoruki–Heck reaction, which is related to the reactions of various alkenes with various aryl halides accompanied with the use of a palladium compound and a suitable base.^33,34^

Here we present an uncomplicated manner for the synthesis of functionalized boehmite nanoparticles with 2-mercaptobenzothiazole and decorated with palladium nanoparticles. The use of this compound, Pd(0)-SMTU-boehmite, as a catalyst in the Suzuki–Miyaura and Mizozuki–Heck cross-coupling reactions was efficient and reusable here.

## Experimental

### Materials and physical measurements

All starting materials were purchased from Merck and Aldrich and used without further purification. An electrothermal device 9100 was used to detect the melting point of the samples. Fourier transform infrared (FT-IR) spectra were recorded with a VRTEX 70 model BRUKER FT-IR spectrophotometer using KBr pellets. Thermogravimetric analyses (TGA) was recorded by using a NETZSCH simultaneous thermal analysis device under an air atmosphere in the temperature range of 30–800 °C and at a heating rate of 10 °C min^−1^. SEM, EDS and EDS-mapping analysis were performed by using a FESEM-TESCAN MIRA3 scanning electron microscope. A TEM Philips EM 208S, 100 kv, transmission electron microscope was used to obtain TEM images. Cu Kα radiation at 40 kV and 30 mA using a PW1730 instrument from Philips Company was used for obtaining the powder XRD patterns of the catalyst. The BET test was performed by using a BELSORP MINI II instrument.

### The (Pd(0)-SMTU-boehmite) nanocatalyst synthesis

At the starting point, a solution of sodium hydroxide, NaOH, (4.867 g) in 30 mL pure water was added to to a 100 mL balloon containing a solution of aluminum nitrate nonahydrate, Al(NO_3_)_3_·9H_2_O, (15 g) in 20 mL of pure water. Then the obtained milk mixture was placed in an ultrasonic bath for 3 h at 25 °C for better and effective mixing. The obtained precipitated particles were washed several times with deionized water and kept in an oven at 220 °C for 4 h.^35^ To obtain the functionalized boehmite nanoparticles, after the boehmite nanoparticles were dispersed in toluene (50 mL) using ultrasound for 30 minutes, 2.5 mL (3-chloropropyl)triethoxysilane (CPTES) was added to it. Stirring of the mixture of the reaction was carried out at 40 °C and this stirring was continued for 8 hours until nanoparticles (nPr-Cl-boehmite) were synthesized. Then these nanoparticles were washed several times with ethanol, and then dried at ambient temperature.

**Scheme 1:**
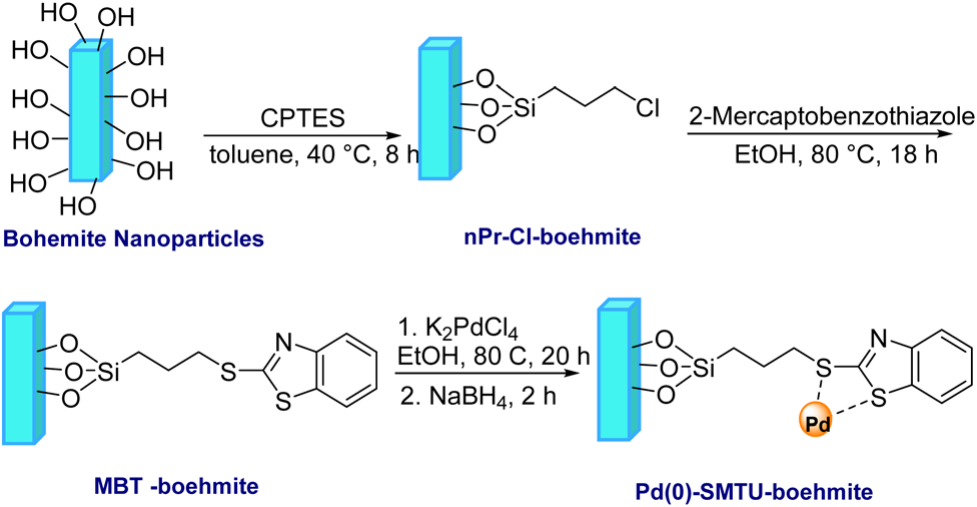
Schematic illustration of the preparation of Pd(0)-SMTU-boehmite nanoparticles.

After that, 2-mercaptobenzothiazole (2.5 mmol) was added to 1 g of freshly dispersed nPr-Cl-boehmite in 50 mL EtOH using a sonicator for 20 minutes. The reaction mixture was continuously stirred at 80 °C for 18 hours. Following this, the suggested (MBT-boehmite) nanoparticles were obtained by filtering, washing with ethanol and drying at ambient temperature. Then, 0.25 g of potassium tetrachloropalladate (K_2_PdCl_4_) was added to 0.5 g of synthesized MBT-boehmite, which was freshly dispersed in ethanol (25 mL) for 20 min using a sonicator. After 20 hours of stirring of the reaction mixture at 80 °C, 0.11 g of NaBH_4_ was added to it and stirring was continued for 2 hours. Finally, the proposed nanoparticles (Pd(0)-SMTU-boehmite) after filtering, washed with ethanol and dried at ambient temperature. Scheme 1 shows the procedure for the nanoparticles (Pd(0)-SMTU-boehmite).

### Catalytic activity in the Suzuki–Miyaura reaction

**Scheme 2:**
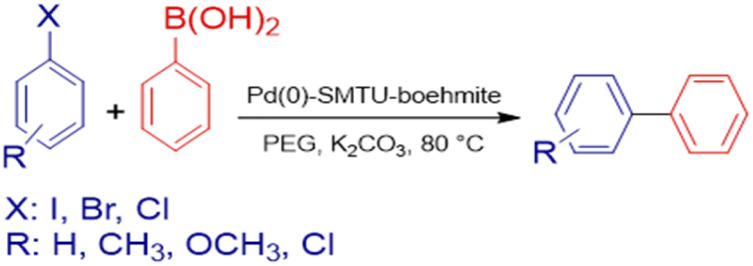
Suzuki–Miyaura coupling reaction of aryl halides and phenylboronic acid catalyzed by Pd(0)-SMTU-boehmite.

The mixture of aryl halide (1.0 mmol), aryl boronic acid (1.2 mmol), potassium carbonate (1.5 mmol), Pd(0)-SMTU-boehmite catalyst (5 mg) and PEG (polyethylene glycol) in a sealable glass tube was stirred at 80 °C for the desired time. The reaction progress was checked by TLC and on completion of the reaction, the catalyst was filtered off and the product was purified. Scheme 2 shows the Suzuki coupling reaction catalyst by Pd(0)-SMTU-boehmite nanocatalyst.

### Catalytic activity in the Mizoroki–Heck reaction

**Scheme 3:**
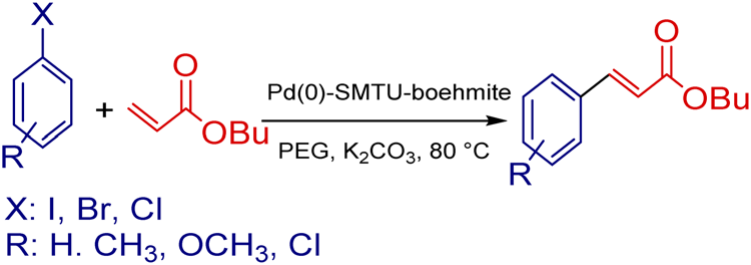
Mizoroki–Heck cross-coupling reaction of butyl acrylate and aryl halides in association with Pd(0)-SMTU-boehmite.

For the Heck reaction, the reaction mixture of aryl halide (1.0 mmol), butyl acrylate (1.2 mmol), 3 mmol K_2_CO_3_, Pd(0)-SMTU-boehmite catalyst (5 mg) and PEG (polyethylene glycol) in a sealable glass tube, was stirred at 80 °C for the desired time. The reaction progress was checked by TLC and on the completion of the cross-coupling reaction, after the addition of hot distilled water to the reaction mixture, the catalyst was filtered off and the product was purified. The relevant product was extracted by using a 1 : 1 mixture of water and ethyl acetate. Scheme 3 shows the Mizoroki–Heck cross-coupling reaction catalyst by Pd(0)-SMTU-boehmite nanocatalyst.

### Sample NMR spectra of some products of Heck and Suzuki reactions

Biphenyl: ^1^H-NMR (300 MHz, CDCl_3_): *δ* 7.71 (dd, 4H, ^3^*J* = 5.7 Hz, ^4^*J* = 3.3), 7.54 (dd, 4H, ^3^*J* = 5.6 Hz, ^4^J = 3.3), 7.29 (m, 2H); ^13^C-NMR (75 MHz, CDCl_3_): *δ* 139.0, 132.1, 128.6, 122.1 ppm.

4-nitro biphenyl: ^1^H-NMR (300 MHz, CDCl_3_): *δ* 8.31 (d, 2H, *J* = 8.8 Hz), 7.75 (d, 2H, *J* = 8.8 Hz), 7.63 (m, 2H), 7.49 (m, 3H); ^13^C-NMR (75 MHz, CDCl_3_): *δ* 147.75, 147.21, 138.90, 129.28, 129.04, 127.92, 127.50, 124.23 ppm.

3-phenyl acrylic acid butyl ester: ^1^H-NMR (300 MHz, CDCl_3_): *δ* 7.63 (d, 1H, *J* = 16.0 Hz), 7.41–7.49 (m, 2H), 7.29–7.39 (m, 3H), 6.39 (d, 1H, *J* = 16.0 Hz), 4.22 (t, 2H, *J* = 6.7 Hz), 1.69 (m, 2H), 1.49 (m, 2H), 0.97 (t, 3H, *J* = 7.3 Hz) ppm; ^13^C-NMR (75 MHz, CDCl_3_): *δ* 166.84, 143.14, 136.39, 133.49, 129.38, 119.48, 64.70, 31.10, 19.40, 13.77 ppm.

3-(2′-Methoxyphenyl)acrylic acid *n*-butyl ester: ^1^H-NMR (300 MHz, CDCl_3_): *δ* 8.00 (d, 1H, *J* = 16.1 Hz), 7.52 (m, 1H), 7.35 (m, 1H), 6.95 (m, 2H), 6.54 (d, 1H, *J* = 16.2 Hz), 4.21 (t, 2H, *J* = 6.9 Hz), 3.90 (s, 3H), 1.68 (m, 2H), 1.44 (m, 2H), 0.97 (t, 3H, *J* = 6.2 Hz) ppm; ^13^C-NMR (75 MHz, CDCl_3_): *δ* 167.78, 158.47, 140.14, 131.51, 129.07, 123.63, 120.82, 118.98, 111.26, 64.42, 55.60, 30.96, 19.35, 13.90 ppm.

3-(4′-methylphenyl)acrylic acid *n*-butyl ester: ^1^H-NMR (300 MHz, CDCl_3_): *δ* 7.66 (d, 1H, *J* = 15.8 Hz), 7.39 (m, 2H), 716 (m, 2H), 6.40 (d, 1H, *J* = 15.9 Hz), 4.21 (t, 2H, *J* = 6.7 Hz), 2.38 (s, 3H), 1.69 (m, 2H), 1.44 (m, 2H), 0.97 (t, 3H, *J* = 7.35 Hz) ppm.

## Results and discussion

### Preparation and characterization of the catalyst

After designing and manufacturing the Pd(0)-SMTU-boehmite nanocatalyst, whose synthesis method is shown in Scheme 1, the desired catalyst was identified using different techniques such as XRD, SEM, EDS, TGA, BET, ICP-OES and FT-IR.

**Fig. 1:**
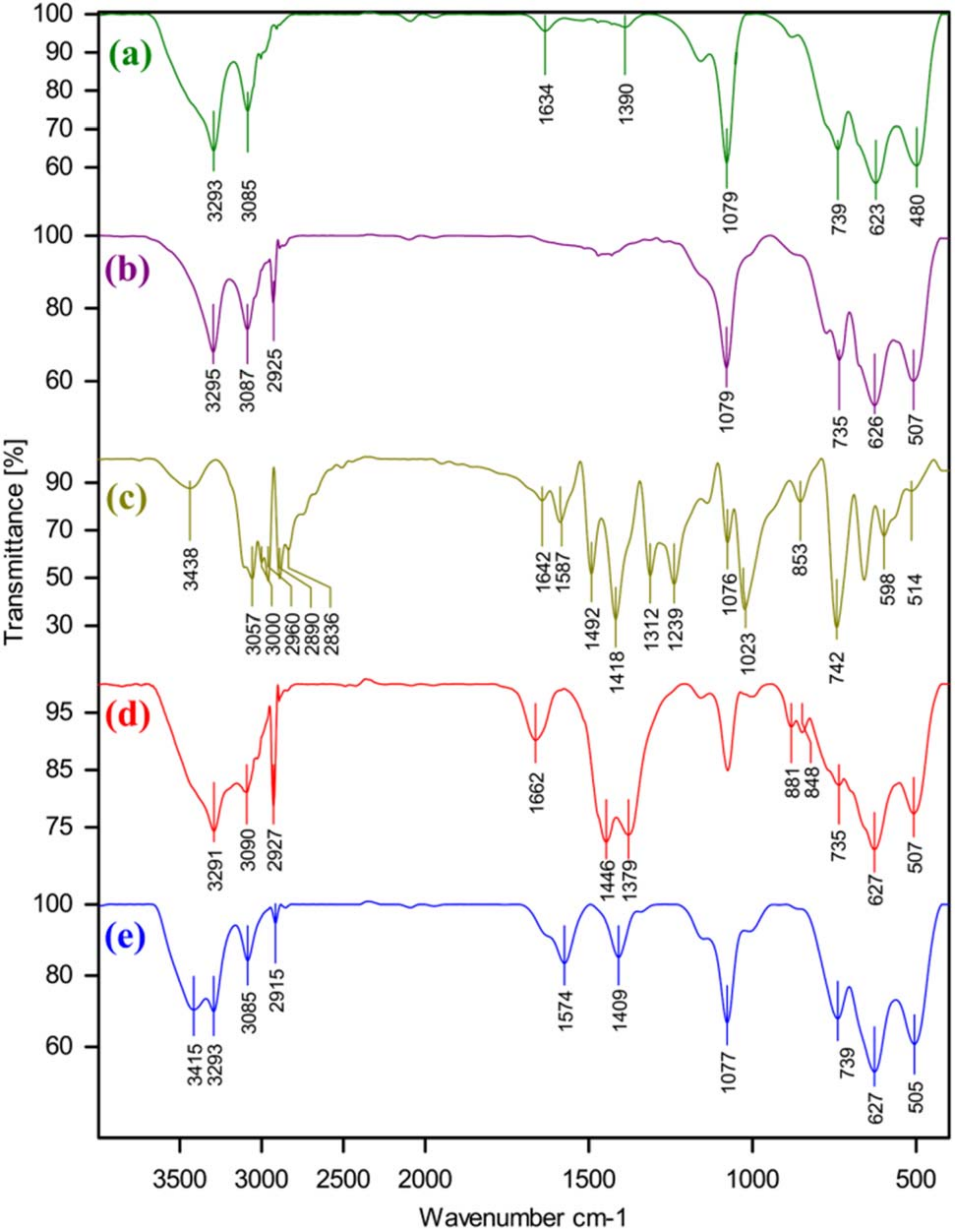
FT-IR spectra for boehmite (a), nPr-Cl-boehmite (b), 2-mercaptobenzimidazole (Bimz) (c), boehmite@nPr-Cl@2-mercaptobenzimidazole (d), and Pd immobilized on boehmite@nPr-Cl@2-mercaptobenzimidazole.

Fig. 1 shows IR spectrum of all parts that participate in the synthesis of Pd immobilized on boehmite@nPr-Cl@2-mercaptobenzimidazole. The boehmite FT-IR spectra (a), nPr-Cl-boehmite (b), 2-mercaptobenzimidazole (Bimz) (c), and boehmite@nPr-Cl@2-mercaptobenzimidazole (d) Pd immobilized on boehmite@nPr-Cl@2-mercaptobenzimidazole (e). Evidence for the occurrence of the synthesized boehmite nanoparticles is the observation of intense vibrational bands at 3293 and also at 3085 cm^−1^ in the synthesized boehmite nanoparticle infrared spectra, due to asymmetric and symmetric vibrational frequencies of two surface O–H bonds over the synthesized boehmite nanoparticles (FT-IR spectrum a). The peaks appearing at 480, 623 and 735 cm^−1^ in FT-IR spectra a, b, d and e arise from the Al–O bond stretching vibration. The existence of anchored trimethoxysilane (3-chloropropyl) can be identified by the vibrational mode caused by the stretching of C–H bonds that occur at 2925 cm^−1^ and stretching vibration mode of O–Si which is present at 1079 cm^−1^ in the nPr-Cl-boehmite FT-IR spectrum. (FT-IR spectrum b). Along with C–C and CH bending vibrations of the compound, the strong vibrational bands at 1012, 1033, and 1076 cm^−1^ are attributed to N–C=S groups. The bands formed at 2640 and 2480 cm^−1^ with very low intensity are attributed to the optical bands of aromatic C–N fundamental frequencies at 1319 and 1244 cm^−1^. The bands detected at 1492 and 1587 cm-1 correspond to the C=C stretching mode. The sharp bands detected at 1418 cm^−1^ belong to CC, CN and CH bending vibrations. Also, the band detected at 1642 cm are in agreement with the C=N vibrational mode. The strong peaks at 1023 and 1076 cm^−1^ are assigned to the N–C=S group. The strong peak at 742 cm^−1^ is assigned to the out-of-plane mode of C–H stretching vibration. Besides, the sharp absorption band at 668 cm^−1^ is associated with the C–S stretching vibrations.

Furthermore, the absorption band at 1662 cm^−1^ corresponds to the C=N bond stretching vibration, while the sharp band at 1446 cm/1 corresponds to the stretching vibration of the C–N aromatic seen in the Pd(0)-SMTU-boehmite nanocatalyst FT-IR spectra (spectrum d) and is related to the linked 2-mercaptobenzimidazole. The above evidence shows that 2-mercaptobenzimidazole is supported on boehmite and the FT-IR spectrum of Pd immobilized on boehmite@nPr-Cl@2-mercaptobenzimidazole displays the expected bands, including a distinct band due to the C=N stretching vibration that occurred at high frequency compared to that of boehmite@nPr-Cl@2-mercaptobenzimidazole.

**Fig. 2:**
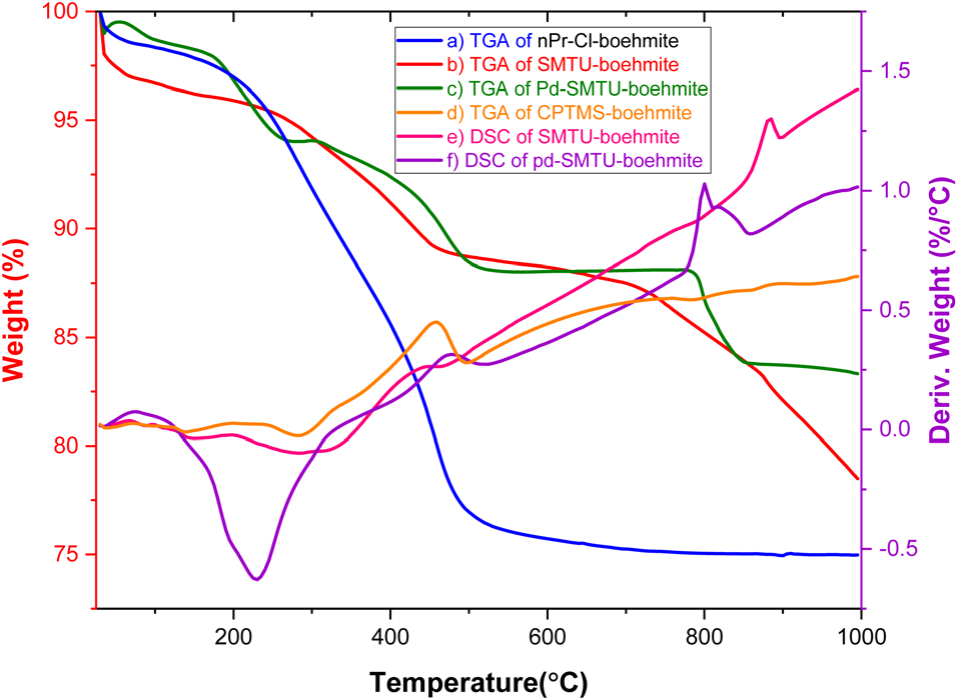
TGA and DSC diagrams of nPr-Cl-boehmite (a–d) SMTU-boehmite (b and e) and Pd(0)-SMTU-boehmite (c and f).

The TGA diagram of nPr-Cl-boehmite, 2- SMTU-boehmite and Pd(0)-SMTU-boehmite is presented in Fig. 2. This graph compares the weight loss in terms of temperature increase for three stages of catalyst synthesis. The low weight loss (9.39%) seen in the first curve (nPr-Cl-boehmite) is attributed to the removal of organic groups attracted to the surface of boehmite and absorbed solvents. In the second graph, the weight loss has decreased to 7.65%, which shows that 2-mercaptobenzimidazole is well placed on the boehmite surface. The last curve is related to catalyst Pd(0)-SMTU-boehmite, whose mass loss is relatively less than that of other prepared materials which indicates successful anchoring of nanoparticles on the boehmite surface, and also displays enhanced thermal stability as compared to SMTU-boehmite, which can be attributed to the increased thermodynamic stability of the nanoparticles.

**Fig. 3:**
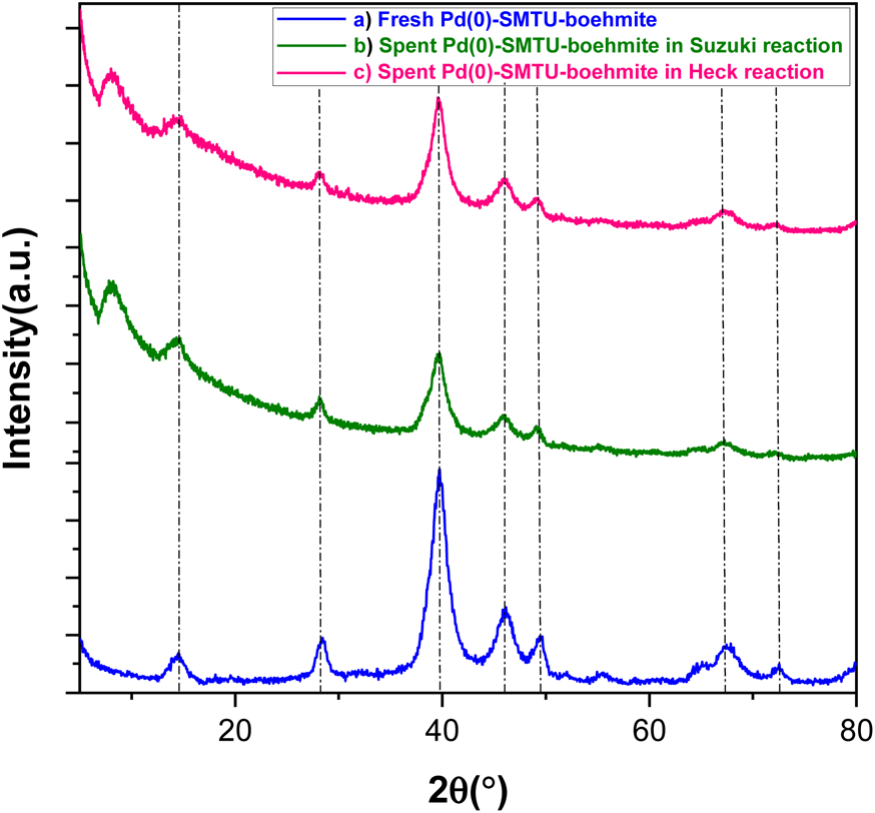
XRD patterns of (a) fresh Pd(0)-SMTU-boehmite and the recovered catalyst in Suzuki (b) and Heck (c) reactions.

The XRD technique was used to determine the structural pattern and check the structural order of boehmite nanoparticles (Fig. 3). The peaks appearing in Fig. 3 show well the synthesis of boehmite nanoparticles.^35^ The peaks in the areas of 39.75, 46.65, 67.85, 82.19 and 87.45 correspond with reflection planes 111, 200, 220, 311 and 222 in the XRD pattern and well show that the palladium zero nanoparticles are stabilized on the boehmite substrate (JCPDS card 46-1043).^36–41^ The presence of these peaks in the catalysts used in Heck and Suzuki reactions shows that the palladium in the sample has maintained its neutrality after the catalytic activity. Calculating the size of nanoparticles using Scherer's formula shows that the size of nanoparticles is about 13 nm.

**Fig. 4:**
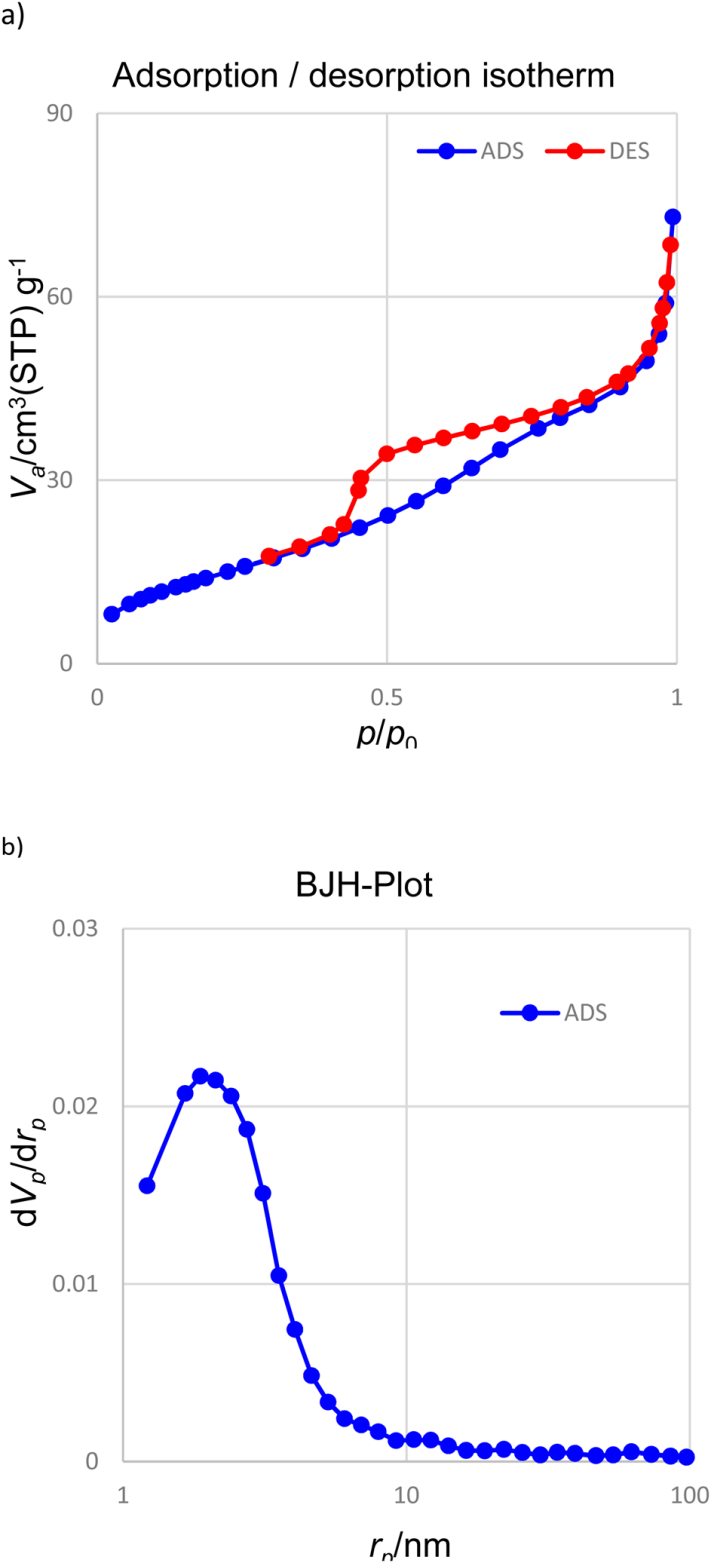
(a) N_2_ adsorption–desorption isotherms and (b) BJH analysis of Pd(0)-SMTU-boehmite.

Fig. 4 shows the nitrogen adsorption–desorption analysis. The calculations related to the BJH diagram show that the average diameter of the holes for the synthesized nanoparticles is 3.89 nm. Based on the BET data (isotherm type IV), the surface area of Pd(0)-SMTU-boehmite nanoparticles is 394.6 m^2^ g^−1^.

**Fig. 5:**
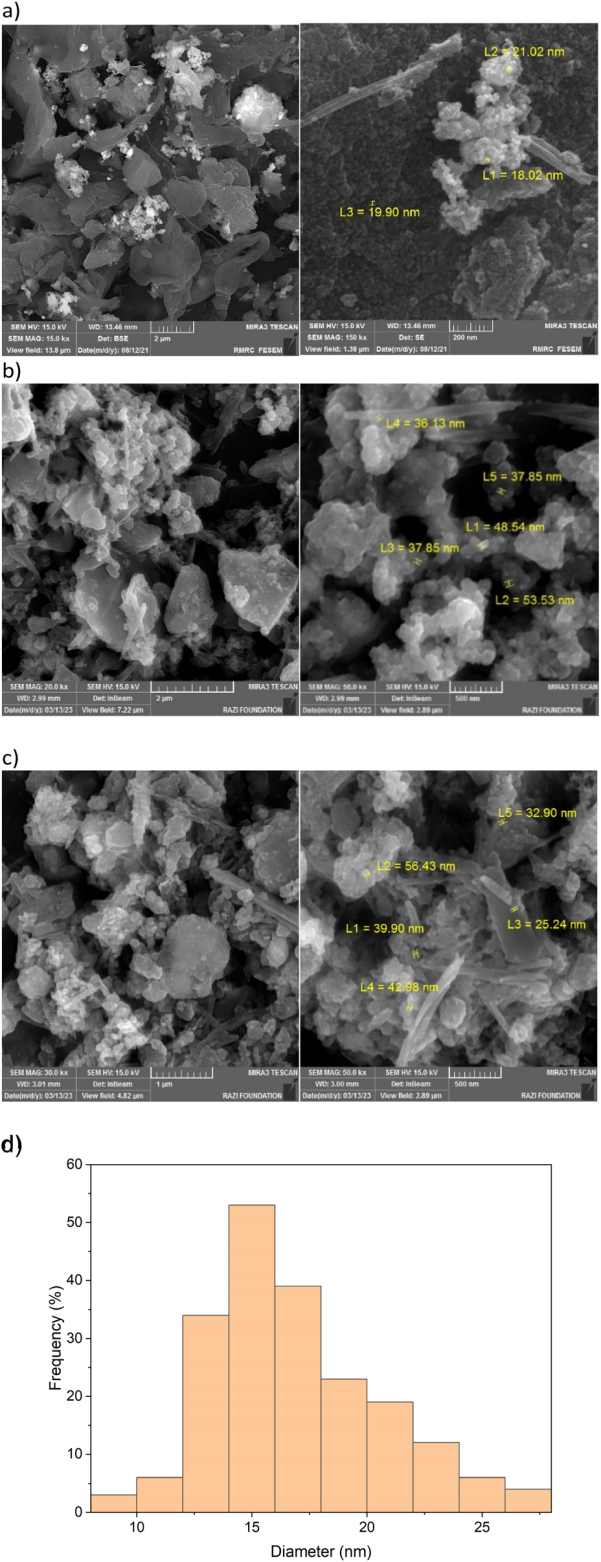
SEM image of the Pd(0)-SMTU-boehmite catalyst, (a) fresh catalyst, (b) and (c) reused catalyst in Suzuki and Heck coupling reactions and (d) particle diameter size histogram distribution.

The structure of the boehmite nanoparticles was confirmed using SEM techniques (Fig. 5a and b). SEM reveals nanorod and nanoplate shape particles with an average diameter of about 16.93 nm.

**Fig. 6:**
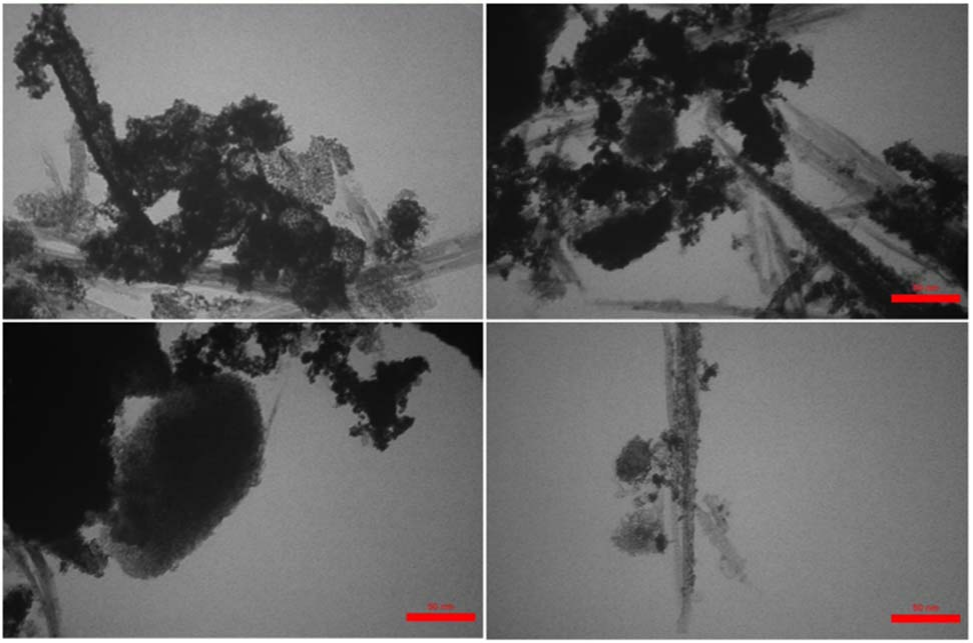
TEM image of Pd(0)-SMTU-boehmite nanoparticles.

Also, the histogram of particle size distribution (PSD) and the corresponding statistical data for the PSD of Pd(0)-SMTU-boehmite are given in Fig. 5d and Table 1, respectively. Morphologies of Pd(0)-SMTU-boehmite nanoparticles as confirmed by TEM (Fig. 6). Transmission electron microscopy (TEM) analysis revealed a combination of nanorods and nanoplates of heterogeneous size.

**Table 1:** Measurement of central tendency (particle number, mean, min, max, median, and standard deviation) for Pd(0)-SMTU-boehmite by using the SEM image

Sample	Mode	Particles	Mean (nm)	Min. (nm)	Max. (nm)	Median (nm)	Standard deviation
Pd(0)-SMTU-boehmite	SEM	199	16.86	8.96	27.93	16.14	3.72

**Fig. 7:**
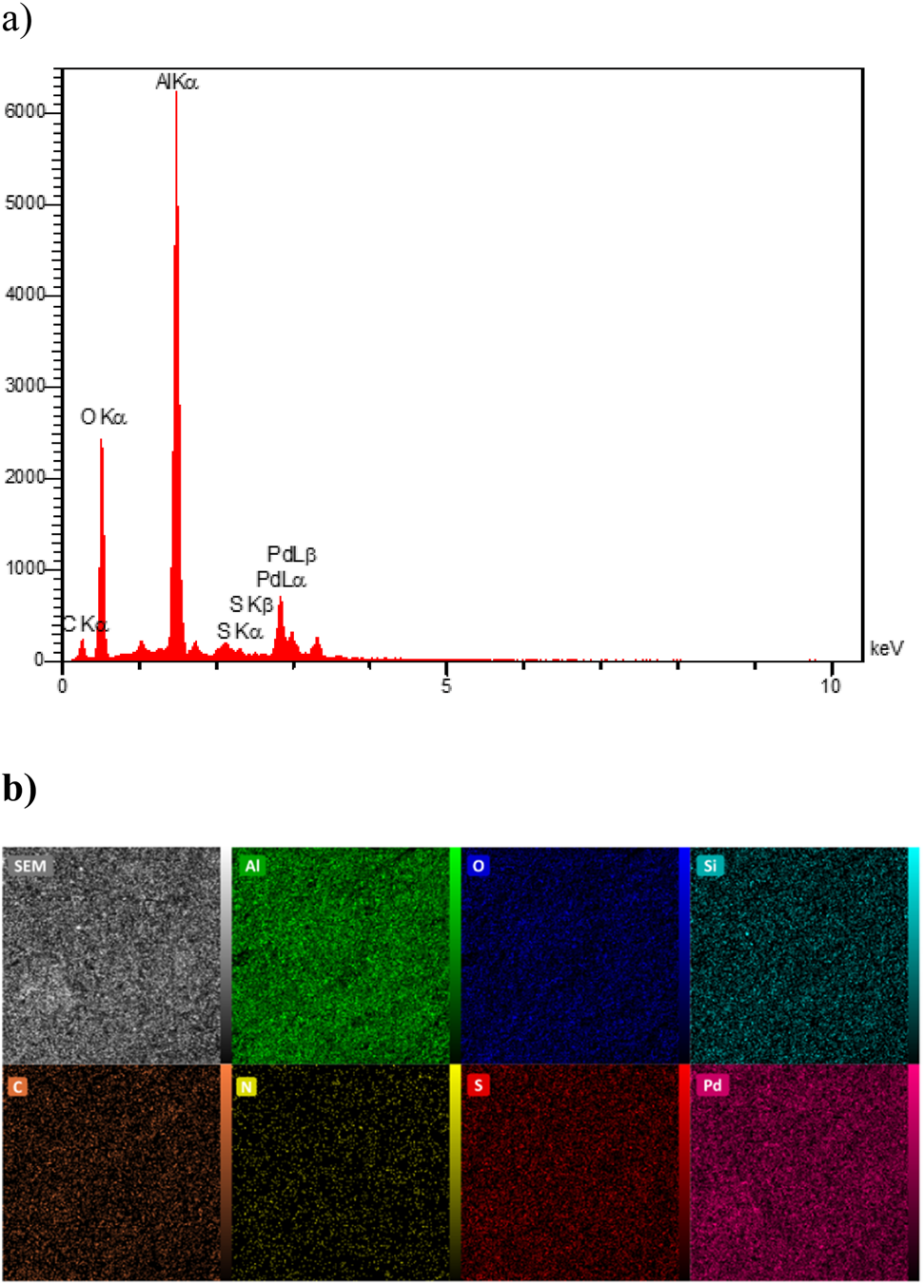
(a) EDS diagram and mapping of Pd(0)-SMTU-boehmite nanoparticles and (b) EDX elemental mapping of Al, O, Si, C, N, S and Pd.

In order to prove the presence of palladium metal on the surface of the functionalized boehmite, the EDS technique was used. The EDS spectrum of Pd(0)-SMTU-boehmite nanoparticles is shown in Fig. 7a. As shown in Fig. 6a, the EDS spectrum of Pd(0)-SMTU-boehmite nanoparticles shows the presence of Al, Al, C, N, O, S, and Si and as well as Pd species in Pd(0)-SMTU-boehmite. The elemental mapping image (Fig. 7b) showed the distribution of all the elements Al, C, N, O, S, Si, and Pd present in Pd(0)-SMTU-boehmite. These findings from the EDAX-mapping analysis contribute to a deeper understanding of the catalyst's composition and can have implications for its catalytic performance. Further investigations into the correlation between element distribution and catalytic behavior could provide valuable insights for optimizing catalyst design and performance in future studies.

### Catalytic studies

After investigating and identifying the catalyst synthesized with boehmite nanoparticles, its catalytic activity in the Suzuki reaction was evaluated. At first, the iodobenzene reaction and phenylboronic acid reagent were selected for the sample reaction and the reaction was investigated under different conditions such as solvent, base, catalyst amount, and temperature. Finally, potassium carbonate (1.5 mmol), 5 mg of catalyst, polyethylene glycol (PEG) solvent, and 80 °C temperature were selected as optimal conditions (Table 2).

**Table 2:** Optimized conditions for the cross-coupling reaction of PhB(OH)_2_ with iodobenzene in association with Pd(0)-SMTU-boehmite

Entry	Catalyst (mg)	Base	Solvent	Temp. (°C)	Time (h)	Yield^a^ (%)
1	—	K_2_CO_3_	PEG-400	80	24	N.R
2	Boehmite	K_2_CO_3_	PEG	80	0.33	N.R
3	5	—	PEG	80	24	N.R
4	5	K_2_CO_3_	CH_3_CN	80	1	>99
5	5	K_2_CO_3_	C_2_H_5_OH	80	0.83	>99
6	5	K_2_CO_3_	DMF	80	0.5	80
**7**	**5**	**K**_**2**_ **CO**_**3**_	**PEG**	**80**	**0.33**	**>99**
8	5	Na_2_CO_3_	PEG	80	0.33	85
9	5	NaOH	PEG	80	0.33	95
10	5	KOH	PEG	80	0.33	>99
11	3	K_2_CO_3_	PEG	80	0.33	85
12	5	K_2_CO_3_	PEG	80	0.33	98
13	10	K_2_CO_3_	PEG	80	0.16	>99
14	5	K_2_CO_3_	PEG	60	0.33	75
15	5	K_2_CO_3_	PEG	80	0.33	98
16	5	K_2_CO_3_	PEG	100	0.33	85

a Isolated yield.

After obtaining the optimal conditions, to expand the application scope of the catalyst in biaryl synthesis through the Suzuki–Miyaura reaction, various types of aryl halides were investigated, which are summarized in Table 3.

**Table 3:** Suzuki–Miyaura coupling reaction catalyzed by Pd(0)-SMTU-boehmite

Entry	Aryl halide	Time (min)	Yield (%)	TON	TOF/h	m.p (°C)
1	Iodobenzene	20	>99	105	315	66–69 (ref. 42)
2	2-Iodotoluene	55	92	97	106	Liquid^43^
3	4-Iodotoluene	25	>99	105	252	45–46 (ref. 44)
4	2-Iodoanisole	60	85	90	90	Liquid^43^
5	4-Iodoanisole	45	90	95	126	84–85 (ref. 45)
6	Bromobenzene	5	>99	105	1260	68–70 (ref. 42)
7	4-Bromoanisole	70	>99	105	90	84–86 (ref. 45)
8	Chlorobenzene	20	30	32	96	69–70 (ref. 42)
9	1-Chloro-4-iodobenzene	40	90	95	142	78–80 (ref. 42)
10	1-Bromo-4-nitrobenzene	50	95	101	121	112–114 (ref. 46)
11	4-Bromobenzonitrile	55	94	100	109	82–84 (ref. 46)

**Scheme 4:**
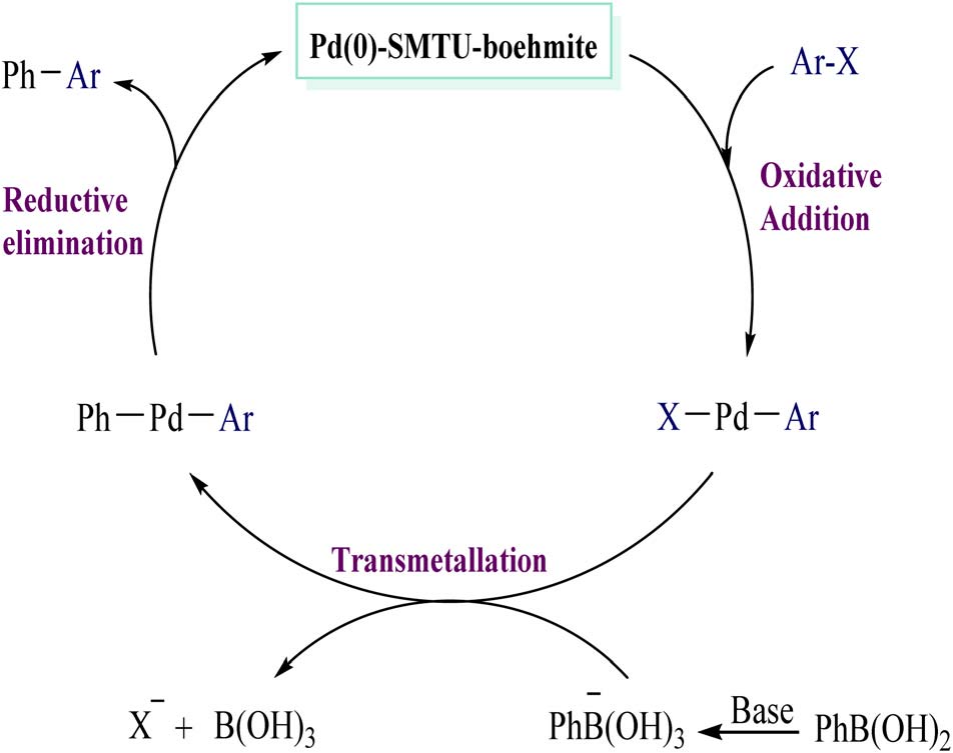
A possible mechanism for the Suzuki–Miyaura reaction.

The Suzuki–Miyaura reaction with an effective catalyst of 0.94 mmol based on the amount of palladium obtained by ICP analysis under normal conditions (Table 2, entries 1–9) shows that the minimum and maximum TON and TOF for the catalyst are about 30–100 and 1200 h^−1^ respectively. Based on previous articles, a proposed route for the synthesis of biaryls is considered and shown in Scheme 4.^28^

Also, to expand the application scope of the Pd(0)-SMTU-boehmite catalyst, the catalyst was used to form a carbon–carbon bond through the reaction of butyl acrylate and different aryl halides. In order to find out how the reaction is carried out, the iodobenzene coupling reaction with butyl acrylate has been selected at a unique reaction point, and different reaction operation conditions such as temperature, amount of catalyst, base and solvent were investigated (Table 4).

**Table 4:** Optimized reaction parameters of the Mizoroki–Heck reaction of iodobenzene in association with Pd(0)-SMTU-boehmite

Entry	Catalyst (mg)	Base	Solvent	Temp. (°C)	Time (h)	Yield^a^ (%)
1	—	K_2_CO_3_	PEG	80	24	N.R
2	Boehmite	K_2_CO_3_	PEG	80	0.33	N.R
3	5	—	PEG	80	24	N.R
4	5	K_2_CO_3_	CH_3_CN	80	0.33	75
5	5	K_2_CO_3_	C_2_H_5_OH	80	0.33	70
6	5	K_2_CO_3_	DMF	80	0.33	90
7	5	K_2_CO_3_	PEG	80	0.33	95
8	5	Na_2_CO_3_	PEG	80	0.33	90
9	5	NaOH	PEG	80	0.33	10
10	5	KOH	PEG	80	0.33	10
11	3	K_2_CO_3_	PEG	80	0.33	85
**12**	**5**	**K**_**2**_ **CO**_**3**_	**PEG**	**80**	**0.33**	**98**
13	10	K_2_CO_3_	PEG	80	0.16	>99
14	5	K_2_CO_3_	PEG	60	0.33	75
15	5	K_2_CO_3_	PEG	80	0.33	98
16	5	K_2_CO_3_	PEG	100	0.33	85

a Isolated yield.

The results show that potassium carbonate, polyethylene glycol solvent, 80 °C temperature, and 5 mg of catalyst were selected as optimal conditions.

After checking the optimal conditions, different derivatives of aryl halides were checked in association with butyl acrylate, and the confirmed results are shown in Table 5.

**Table 5:** Mizoroki–Heck reaction in association with Pd(0)-SMTU-boehmite

Entry	Aryl halide	Time (min)	Yield (%)	TON	TO/h	m.p (°C)
1	Iodobenzene	25	>99	105	252	Liquid^47^
2	2-Iodotoluene	5	95	101	1140	Liquid^43^
3	4-Iodotoluene	6	95	101	1010	Liquid^47^
4	2-Iodoanisole	6	95	101	1010	Liquid^43^
5	4-Iodoanisole	6	97	103	1030	Liquid^47^
6	Bromobenzene	150	15	16	6	Liquid^47^
7	4-Bromoanisole	150	70	74	30	Liquid^43^
8	Chlorobenzene	150	15	16	6	Liquid^42^
9	1-Chloro-4-iodobenzene	10	90	96	576	Liquid^42^
10	1-Bromo-4-nitrobenzene	130	92	97	45	62–64 (ref. 46)
11	4-Bromobenzonitrile	280	94	100	21	44–46 (ref. 46)

**Scheme 5:**
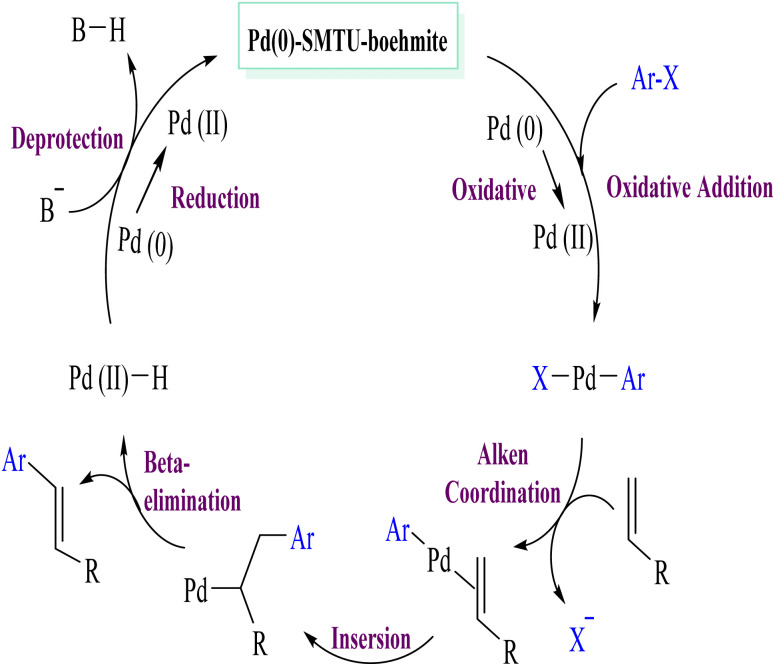
Proposed mechanism of the Mizoroki–Heck reaction involving Pd(0)-SMTU-boehmite.

The results of the Mizoroki–Heck reaction with the conditions in Table 4 show that the minimum and maximum TON values are 16 and 100, respectively. In order to check the progress of the reaction, the calculated reaction TOF and the results presented in the table are in the range of 6 to 1100. In Scheme 5, a proposed mechanism for the Mizoroki–Heck reaction is presented. This mechanism includes an oxidative addition, an insertion step, a beta hydride elimination step, and a reductive elimination step that finally recycles the catalyst and returns to the cycle.^48^

### Catalyst recycling

**Fig. 8:**
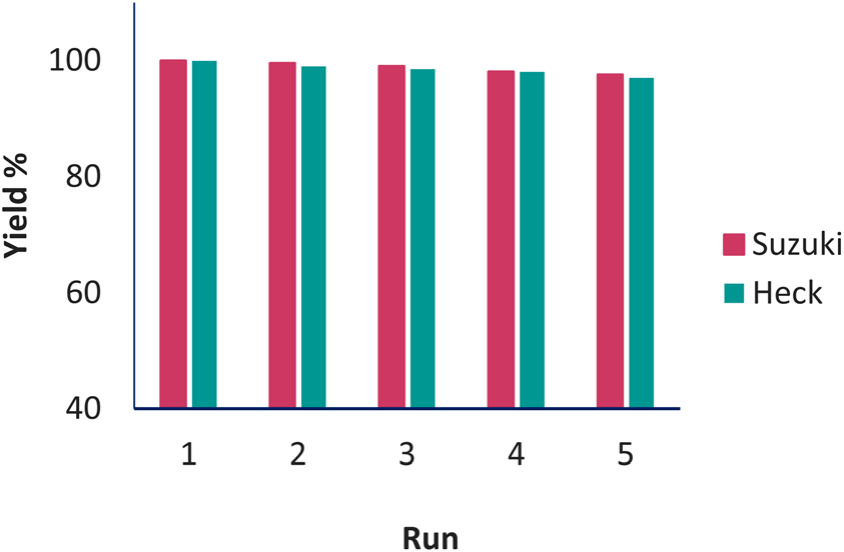
Pd(0)-SMTU-boehmite catalyst recovery.

In catalytic systems, recycling and reuse of catalysts are precious factors that are very important from an economic and environmental point of view for industrial applications. In order to recover and reuse the Pd(0)-SMTU-boehmite catalyst, the iodobenzene C–C coupling reaction along with phenylboronic acid and also with butyl acrylate were selected as a specific reaction for the Suzuki–Miyaura reaction and the Mizoroki–Hack reaction, respectively. After the reaction was complete, the catalyst was filtered. Then the isolated catalyst was washed with ethyl acetate and used for the subsequent steps. As shown in Fig. 8 reusability of the catalyst was achieved 5 consecutive times without the reduction of the catalytic activity. In order to show the structural stability of the catalyst after recycling, the recovered catalyst was characterized using the XRD technique. The recovered catalyst was investigated using XRD and SEM (Fig. 3b and 5b). The SEM image and XRD pattern of the recovered Pd(0)-SMTU-boehmite indicate that this catalyst can be recycled without any change in its structure.

The Pd(0)-SMTU-boehmite catalyst performance in this synthetic method has been compared with that in other previously presented methods for the preparation of biaryl compounds (reaction of phenylboronic acid and iodobenzene) (Table 6).

**Table 6:** Estimating the efficiency of the Pd(0)-SMTU-boehmite catalyst compared to that of some previously reported catalysts in the Suzuki–Miyaura reaction

Entry	Catalyst	Time (min)	Yield (%)	Ref.
1	Pd-NPs@Cu_2_(BDC)_2_DABCO	60	95	49
2	Pd(OAc)_2_	24	95	50
3	Pd-BS-MCM-41	60	91	51
4	Pd-porphyrin@polymer	10	93	52
5	GO-Met-Pd	15	98	53
6	Pd(0)-SMTU-boehmite	20	>99	This work

## Conclusion

Herein, Pd(0)-SMTU-boehmite catalyst nanoparticles were prepared, characterized and used in the creation of carbon–carbon bonds *via* general Suzuki–Miyaura and Mizoroki–Hack reactions. Pd(0)-SMTU-boehmite nanocatalysts were recycled and reused five times consecutively without significant decrease in catalytic activity.

## Conflicts of interest

No declarations of interest are reported by the authors.

## Supplementary Material
